# Improving blood safety using fourth generation HIV ELISA as the screening tool in blood banks – an Indian experience

**DOI:** 10.1186/1471-2334-12-S1-P89

**Published:** 2012-05-04

**Authors:** Sheetal Malhotra, Neelam Marwaha, Karan Saluja, Ratti Ram Sharma

**Affiliations:** 1 Department of Transfusion Medicine, Postgraduate Institute of Medical Education and Research, Chandigarh, 160012, India

## Background

The newer fourth generation HIV ELISA has been shown to have an increased sensitivity compared to third generation ELISA.

### Objectives

To estimate the yield of fourth generation HIV ELISA compared to third generation HIV ELISA assay and to determine HIV seroprevalence among blood donors.

## Materials and methods

This prospective study involved 10200 blood donors – 6800 voluntary donors (3400 – students and 3400 – non students) and 3400 replacement donors. All blood units were tested using third and fourth generation ELISA. All positive & borderline positive samples were confirmed by Western Blot (WB).

## Results

The HIV seroprevalence was estimated to be 1.37/1000 donations with 3^rd^ generation and 3.62/1000 donations with 4^th^ generation ELISA (p>0.05). Of the 17 samples which were 4^th^ generation ELISA positive and WB positive, 11 were positive with 3^rd^ generation ELISA. Fourth generation ELISA tested 19 additional samples positive and 7 samples as possibly positive which were tested negative with 3^rd^ generation ELISA, giving an additional yield of 26 window period units per 10200 donations i.e. 2.5/1000 donations. Of these, 6 were WB positive giving a yield of 0.58 window period units per 1000 donations. Since 2-3 components are prepared from each blood unit, the yield can be increased to 12-18/10200 donations. With an annual donation of nearly 46000 blood units, this will be 54-81 positive samples per 10200 donations.

## Conclusions

Prevention of 54-81 individuals from acquiring new transfusion transmitted HIV infection with the newer fourth generation HIV ELISA assay with better performance and comparable cost is a cost-effective strategy.

